# Living with pain and Parkinson’s developing an understanding of the impact, trajectory and pain management needs: a qualitative interview study protocol

**DOI:** 10.1136/bmjopen-2023-078754

**Published:** 2024-12-26

**Authors:** Jenni Naisby, Leah Avery, Katherine Baker, Mark Parkinson, Annette Hand, Lynn Rochester, Alison Yarnall, Richard Walker, Darren Flynn, Cormac Ryan, Tracy Finch

**Affiliations:** 1 Department of Sport, Exercise and Rehabilitation, Northumbria University, Newcastle upon Tyne, UK; 2 School of Health & Life Sciences, Teesside University, Middlesbrough, UK; 3 Newcastle Hospitals NHS Foundation Trust, Newcastle, UK; 4 Department of Nursing, Midwifery and Health, Northumbria University, Newcastle upon Tyne, UK; 5 Translational and Clinical Research Institute, Newcastle University, Newcastle upon Tyne, UK; 6 Newcastle University, Newcastle upon Tyne, UK; 7 Northumbria Healthcare NHS Foundation Trust, North Shields, UK; 8 Northumbria University, Newcastle upon Tyne, UK; 9 Teesside University, Middlesbrough, UK; 10 Nursing, Midwifery and Health, Northumbria University, Newcastle upon Tyne, UK

**Keywords:** PAIN MANAGEMENT, Parkinson-s disease, QUALITATIVE RESEARCH

## Abstract

**Abstract:**

**Introduction:**

Pain is reported as one of the most troubling symptoms for people with Parkinson’s (PwP); however, the literature exploring their lived experience of pain and how to manage it is limited. Pain affects PwP at all stages of their condition and can fluctuate and change over time. Therefore, it is pertinent to speak to PwP to understand their experiences of pain to inform the development of tailored behavioural interventions to manage pain. How pain interacts with other Parkinson’s symptoms lacks consensus. Gaining a better understanding of this from the perspective of PwP is important to inform interventions. Exploring the behavioural determinants, including the barriers and enablers to pain management from the perspective of PwP, the role of healthcare professionals and impact of other symptoms alongside pain will inform the development of a fit for purpose, pain management toolkit for PwP.

**Methods and analysis:**

A longitudinal qualitative study using semi structured interviews at two time points within an 18-month period will be conducted. PwP living with pain will be purposefully sampled from four NHS sites in the North of England. Data will be thematically analysed with reference to the Theoretical Domains Framework.

**Ethics and dissemination:**

A favourable ethical opinion has been granted by the National Health Service East Midlands-Derby Research Ethics Committee (22/EM/0176) and the NHS Health Research Authority (IRAS ID 316403). Findings will be disseminated via scientific conferences, academic journals, lay summaries and public engagement events.

**STRENGTHS AND LIMITATIONS OF THIS STUDY**

- This study will qualitatively explore people with Parkinson’s pain experience longitudinally, which will develop detailed understanding of the trajectory of pain in Parkinson’s and pain management over time.
- This study will use the Theoretical Domains Framework to inform interview question design and analysis to guide the selection of theory-linked behaviour change techniques in future research.
- This study is recruiting from the North of England, so transferability will need to be considered in the context of location.

## Introduction

 People with Parkinson’s (PwP) describe pain as one of their most troubling symptoms.^1^ They experience pain disproportionately compared with the general older adult population^2^,^4^ with up to 85% of those diagnosed with Parkinson’s reporting pain.^5 6^ Both dopaminergic and non-dopaminergic central nervous system pathways can be affected by the neurophysiological processes of Parkinson’s, and these pathways are important for pain processing.^7^ While there is a growing understanding of the mechanisms of pain in Parkinson’s, there are limited interventions available for PwP who experience pain.^8^ Furthermore, there is a lack of consensus within the literature in terms of whether other Parkinson’s symptoms are associated with pain.^2^ This complex interplay requires greater understanding to develop future successful pain management interventions. PwP express the need for improved pain management strategies to impact positively on physical function and mood.^9^ However, there is an insufficient awareness of pain in Parkinson’s by clinicians,^10^ and limited numbers of healthcare professionals (HCP) currently support pain management in PwP.^9 11 12^ As such, there is an urgent requirement to better understand how pain in Parkinson’s can be better managed.

Parkinson’s is complex, with individuals facing motor symptoms including mobility and balance problems and non-motor symptoms affecting mood and sleep and autonomic disturbance. PwP may be predisposed to pain due to disease processes^7^ and experience multiple types of pain^13^ making this condition challenging.^11^ PwP are unique in having pain alongside a wide range of motor and non-motor symptoms. Furthermore, pain can fluctuate and change in Parkinson’s.^9 14^

Pain management is limited in Parkinson’s.^10^,^1215 16^ A recent systematic review of pharmacological and non-pharmacological therapies for pain and Parkinson’s found that more research is needed for pain management.^8^ Between 50%^12^ and 63%^15^ of PwP reporting pain have not received pharmacological or non-pharmacological treatment. Collectively, there is limited knowledge regarding what type of pain management support is required for PwP and how to implement this. To facilitate this, research is required to understand the impact of pain, its management and the interplay of Parkinson’s symptoms and pain over the disease course. A greater understanding of these areas will guide the development of bespoke pain management interventions, which are currently limited for PwP with pain.^8^ This research is required because pain in Parkinson’s is under recognised and not well understood within clinical practice.^12^,^16^

It is important to understand the experiences of pain and what support PwP need. In one qualitative study,^17^ researchers conducted interviews with 15 PwP and identified musculoskeletal pain as a key problem. While experiences of healthcare were positive, this formed a small part of the findings. An interpretative phenomenological analysis exploring the lived experience of pain (n=4)^17^ reported that PwP experienced high levels of pain, and a disconnect between HCPs and PwP meant pain needs were not addressed. While small sample sizes can be expected in interpretative phenomenological analysis, transferability of findings are limited. Therefore, further qualitative research is required to specifically explore the behavioural determinants, including barriers and enablers, to pain management more broadly.

Participation in physical activity has been found to be negatively influenced by pain in PwP,^18 19^ but is encouraged for PwP as part of overall management. A mixed methods survey of PwP and pain^9^ was conducted to understand the impact of pain in Parkinson’s and to understand current management and preferences for pain management. Findings indicated that pain negatively impacts day-to-day life, and individuals consistently reported the need for support to manage their pain. Qualitative detail was limited given the nature of the survey. As such, there is a considerable gap in terms of understanding the issues faced by PwP and pain, the role of HCPs, how pain is or should be managed and the specific support PwP require.

This qualitative study is part of a broader programme of work that aims to develop a co-created pain management toolkit for PwP and a matched clinician training package. This qualitative study is key to inform subsequent co-design workshops to develop a pain management toolkit. This co-created pain management toolkit and clinician training package aims to support HCPs to better understand pain in PwP, provide personalised support and improve how PwP manage their pain. This will have potential for high health and socioeconomic impact through empowering and providing access to support for PwP to develop the confidence, skills and knowledge to manage their pain and its multifactorial impact.

In summary, there is a lack of qualitative literature exploring pain and Parkinson’s. Consequently, there are few evidence- and theory-informed interventions for the management of pain in Parkinson’s. Due to the changing trajectory of both pain and Parkinson’s, exploring experiences over time is warranted. There is an urgent need to develop an understanding of pain in Parkinson’s to provide support to both HCPs and PwP on management of pain.

## Methods and analysis

### Study aims and objectives

This qualitative interview study aims to: (i) understand the experiences of living with pain from the perspective of PwP and (ii) identify the behavioural determinants, including barriers and enablers to effectively managing pain and related symptoms. These aims will be achieved through the following objectives:

- Explore the experiences of living with pain and the motor and non-motor symptoms of Parkinson’s and any changes over time.
- Explore the impact, meaning, management and changes in pain for PwP over time.
- Explore the role of HCP support for pain and PwP.
- To use the capability’, ‘opportunity’, ‘motivation’ and ‘behaviour’ (COM-B) model and Theoretical Domains Framework (TDF) to identify behavioural determinants, including barriers and enablers to pain management in PwP.

### Theoretical framework

This study will adopt a constructivist paradigm^20^ that considers that people construct their own realities; therefore, the qualitative methods used will allow for these realities to be explored to develop an understanding of individual experiences. The Behaviour Change Wheel^21^ (BCW) and the embedded TDF^22^ will be used to generate and analyse data that will inform selection of theory-informed intervention content for inclusion in a behavioural intervention. The BCW summarises 19 frameworks of behaviour change and offers a systematic approach to identifying relevant intervention functions based on an in-depth behavioural analysis.^22^ At the centre of the BCW is the COM-B model and the TDF maps onto the COM-B system.^23^ The TDF is the result of a multidisciplinary expert consensus approach, derived from 33 behavioural theories and 128 key constructs that have been organised into 14 theoretical domains based on commonalities.^24 25^ These 14 domains (eg, knowledge is linked with the capability component of the COM-B system) provide broad explanations of why a target behaviour may (enablers) or may not (barriers) be performed.

The BCW and embedded TDF will support the identification of specific behavioural domains and will guide the selection of theory-linked behaviour change techniques for inclusion in the future co-designed intervention.

The analysis of data from interviews with reference to the TDF will be flexible, so as not to eliminate data that does not naturally correspond to domains within the TDF. This combination of an inductive and more structured approach to analysis will allow for a broader exploration of participants’ experiences. Although behavioural factors are the key focus for gaining the necessary understanding of the lived experiences of PwP for the development of appropriate interventions, it is acknowledged that wider, non-behavioural factors are also likely to be relevant to PwPs experiences and responses.

It is important that interventions are developed following identification of target behaviours; however, traditionally this approach (without the use of frameworks such as the BCW) has been limited.^23^ Due to there being limited interventions in the context of pain management and Parkinson’s, it is important to identify the antecedents of target behaviours that will positively influence pain management.

### Study design

A longitudinal qualitative study using two semi structured one-to-one interviews will be conducted. Longitudinal interviews have been selected because pain for PwP changes over time.^14 26^ Longitudinal interviews are advocated when change is the key focus, and from a pain management perspective, it is important to understand what is required, and when.^27^ Furthermore, longitudinal interviews allow a deeper understanding of how and why change occurs and what contributes to change reported over time.^28^ Low self-efficacy and fear avoidance are risk factors for the development of chronic pain, and it will be important to explore this in PwP.^29^ As pain is a subjective, biopsychosocial experience, interviews will facilitate a holistic exploration from the individuals perspective. The second interview will allow exploration of key areas identified from the analysis from the first interview, for example, fluctuations in a pain experience and the impact and management of this.

### Recruitment and participants

#### Sampling

A purposeful sample of PwP who experience pain (disease duration, gender, age, stage of Parkinson’s, NHS location) will be recruited. A variation is required given the lack of consensus in the literature regarding stage of Parkinson’s, disease duration and pain. Pain has been demonstrated to be problematic across the trajectory of the condition; therefore, this study aims to capture a range of experiences from PwP. Recruiting from multiple sites allows a detailed understanding of current management and healthcare approaches to pain across care teams. Characteristics of the sample will be monitored, and recruitment targeted accordingly. While it is understood the merits of a validated pain assessment tool, within the study, we aim to capture the multifactorial subjective experience of pain in individuals own words and chose not to classify pain in advance.

We aim to recruit up to 30 PwPs to be interviewed at two time points within an 18-month time period. However, an exact sample size cannot be determined a priori, and the context and ongoing findings of the study will inform final numbers.^30^ Recruiting up to 30 participants aims to achieve maximal variation in the purposeful sample described above. Longitudinal interviews will aim to generate a large amount of in-depth data; therefore, fewer participants are required than if interviewing at one time point. The depth surrounding living with pain, changes that occur and pain management support needs are a key target of this research.

Recruitment will take place at four NHS Parkinson’s services in the North of England. These four Parkinson’s clinics cover a diverse geography including rural, urban and different deprivation index areas in close proximity. PwP that meet the eligibility criteria will be made aware of the study during clinic visits and provided with a patient information sheet (PIS). If there is limited recruitment or the purposeful sample needs to target specific characteristics, then clinical staff at NHS sites will screen individuals notes for eligibility and post an invitation letter and PIS.

##### Inclusion criteria

- A diagnosis of Parkinson’s according to the UK Brain Bank Criteria.
- Report experiencing pain when living with Parkinson’s frequently for 3 months or more that can interfere with daily life
- Capability to provide informed consent
- Adequate verbal communication skills,
- Aged 18 years or over
- Able to converse in English.

##### Exclusion criteria

- Person has features indicating another type of degenerative parkinsonism, for example, progressive supranuclear palsy
- Evidence of significant cognitive impairment

### Data collection

PwP will be invited to participate in two semi structured interviews within an 18-month time period. Interviews one and two will be conducted at least 6 months apart. Participants will be given a choice regarding how they would like their interviews to be conducted. These options include a face-to-face interview at their home, a telephone interview or interview via a virtual platform (eg, Microsoft Teams). It is envisaged that each interview will take up to 1 hour, although this may vary depending on the individual. All interviews will be audio-recorded.

The topic guide will aim to capture the individuals’ experiences of living with Parkinson’s and symptoms associated with this (see online supplemental table 1). Qualitative exploration will facilitate an understanding of the unique experiences of living with pain and motor and other non-motor symptoms. The study also seeks to understand current management approaches for pain, barriers and enablers associated with engaging with these and key behaviours that should be the target for interventions to enable health behaviour change. Previous literature has identified HCP involvement in Parkinson’s pain management^9^ but has highlighted that limited support is offered/available;^12^ therefore, the role of HCPs and other sources of support will be explored. The findings from interview one will inform the content of the topic guide for interview two. This will enable a deeper understanding of the experience of pain over time that is pertinent to the individual PwP. In addition, participants will be asked to reflect on how their pain has manifested and changed over time and discuss any changes that have occurred since their last interview that will provide insights into how flexible and adaptable any intervention developed should be. The order of questions will be flexible to allow natural conversation.^31^

Prior to the second interview, a COM-B self-evaluation questionnaire will be emailed or posted (depending on participant preference) to individuals to complete prior to the second interview. Individuals will return these via email or post, whatever their preference. The outcome of individual questionnaires will inform questioning and discussions during the second interview.

The study is supported by a patient and public involvement (PPI) advisory group, which includes three PwP and one carer. Qualitative interviews have been piloted with this group, and feedback regarding questions will inform interviews with PwP. The COM-B self-evaluation questionnaire will be piloted with this group and some amendments made, before being sent to PwP.

Each of the TDF domains will not all be considered with specific questions, and instead more general open questions will be incorporated into a topic guide.^32^ General open-ended questions regarding the experience of Parkinson’s, pain and management will be included. However, due to the limited pain management interventions for Parkinson’s, thorough understanding of key behavioural determinants is also required to inform future intervention development. Pain is a biopsychosocial experience^33 34^ with both physical and psychological factors being relevant to consider. Cognitive and affective dimensions form a key part of a pain experience; therefore, individuals’ beliefs and emotions should be considered when exploring behaviours related to pain and its management, especially as low self-efficacy and fear avoidance can negatively impact a pain experience and lead to chronicity.^29^ Questions focusing around these will be included within the topic guide, informed by COM-B^23^ for interview two. Questions proposed within the second interview aim to map to the COM-B questionnaire so that areas important to the individual can be further explored. Through analysis of the first interview, key behavioural areas may also be found, which can be explored in detail within interview two.

Data collection has begun in February 2023 and will conclude in July 2024.

### Data analysis

All interviews will be audio recorded and transcribed verbatim. Interviews will be thematically analysed.^35^ with reference to the TDF. As the proposed study is exploring the experience and impact of pain over time alongside target behaviours for pain management, inductive and deductive approaches will be used. The TDF will be valuable in supporting identification of barriers and enablers to pain management and identifying behavioural determinants to inform the future toolkit.

NVivo software will be used for data management, and all data will be coded and organised using this software. Analysis will be carried out, while data collection is ongoing. This aims to allow reflection and inform future interviews. Two researchers will independently code the first four transcripts. They will then meet to discuss codes and develop an initial thematic framework. Remaining transcripts will be split and analysed by one of the researchers. The researchers will meet every five transcripts to discuss analysis and identification of any new codes. There will be regular meetings among the wider research team (authors) for peer debrief to discuss emerging themes. Peer debriefs will be key as the range of backgrounds from the authors (Psychology, Medicine, Nursing, Physiotherapy) will ensure multiple perspectives to inform data analysis. The study PPI group will be consulted on the developing themes and how this informs future data collection.

Within case and cross case longitudinal analysis will be carried out^36^ following completion of the two interviews per participant. This aims to capture changes in symptoms, pain, and health behaviour over time.

### Patient and public involvement

JN was awarded a Parkinson’s UK Research Involvement Award in February 2020. Part of the work conducted was a focus group discussion with five PwP/carers. The role of qualitative research was discussed and PwP discussed the importance of capturing individuals’ stories regarding pain and Parkinson’s. For the duration of the study (and fellowship), there will be a steering group of four PwP/carers. This group will meet quarterly to discuss the management and undertaking of the research. For this study, the group will advise and feedback on the research including piloting of the interview guide and COM-B questionnaire, theme development and dissemination including lay summaries. The PPI advisory group will be paid following UK National Institute for Health Research guidance^37^

### Ethics and dissemination

This study has had approval from the National Health Service (NHS) East Midlands-Derby Research Ethics Committee (22/EM/0176) and NHS Health Research Authority (IRAS ID 316403).

The interview could cause some participants distress due to focusing on experiences of pain and the impact of this on the individual. The research team would approach discussion of pain sensitively and empathetically. Participants can stop the interview at any point. Participants will be advised that they can discuss any concerns with their Parkinson’s NHS service. The PIS also gives details of the Parkinson’s UK support helpline.

We will work alongside PwP in the steering group to provide lay summaries of the research. A blog of the stages of the findings will be shared with Parkinson’s UK. The authors include clinicians who will support dissemination through their networks. Videos may be produced with support from visual storyboarding to share findings. The work will be published in journals and presented at conferences targeting a range of HCP and specialties including pain, movement disorders and geriatrics. Results may be shared on social media and via public engagement.

## Discussion

To our knowledge, this is the first study to qualitatively explore people with Parkinson’s pain experience longitudinally. This will develop in-depth understanding if pain in Parkinson’s and its management changes over time and the impact this has on individuals. This study will also use the TDF to explore barriers and enablers to pain management. Understanding key behavioural determinants is key to develop future pain management interventions for people with Parkinson’s living with pain, which to date have been limited.

## supplementary material

10.1136/bmjopen-2023-078754 online supplemental file 1
