# Supplementary material for: Living with pain and Parkinson’s developing an understanding of the impact, trajectory and pain management needs: a qualitative interview study protocol
Source: BMJ Open. 2024 Dec 26;14(12):e078754. doi: 10.1136/bmjopen-2023-078754 (PMC11683903; doi:10.1136/bmjopen-2023-078754)
Supplement: online supplemental file 1 [file bmjopen-14-12-s001.docx]

**Supplementary Table 1: Topic Guide**

Between interview one and two, participants will be provided with the COM-B Self Evaluation Questionnaire, which covers Capability, Opportunity and Motivation. Areas noted as relevant to that individual will be used to inform interview two questions that focus on COM-B areas. Data analysis of individuals transcripts from interview 1 will also inform key areas to focus within interview 2. Participants will be asked to reflect on how their pain has behaved over time and any changes that have occurred since the last interview within the topic guide areas.

| **Area of questioning** | **Interview 1 Example question areas/prompts** | **Interview 2 Example question areas/prompts** |
| --- | --- | --- |
| Background | - About self - Family, friends - Occupation, hobbies |  |
| Experience of living with Parkinson’s | - Symptoms leading up to diagnosis - When diagnosed/experience of diagnosis - Symptoms experienced and the impact - Medication/management of Parkinson’s - Support networks | Changes in Parkinson’s symptoms and management since we last met  (to understand context) |
| Experience of pain | - Past/current experience - Day to day impact - Description of pain - The impact of pain alongside other symptoms | Have there been changes in pain since we last met  The impact of pain alongside these other symptoms  Description of pain  *Based on COM-B feedback:*  Knowledge of pain (*Psychological capability)*   - Understanding - Meaning - Why individual feels experiencing pain - What individual thinks can be done about pain |
| Management of pain | - Feelings about management - Current approaches (if applicable) (e.g. non pharmacological, pharmacological, psychological) - Challenges - Advice given - What the individual would like from pain management | Changes in management approaches and what has informed this  *Based on COM-B feedback:*  Explore skills *(Physical Capability, Psychological Capability)*   - Do you know how to manage your pain - How easy/difficult are day to day activities due to pain - What skills needed to manage pain that meets your needs - What affects your ability to manage your pain? Why?   Explore beliefs *(Reflective motivation)*   - How difficult or easy is it for you to do x (informed by interview) - How confident do you feel about x e.g. day to day activities with pain (informed by interview)   Probe: maintenance of this   - Problems/difficulties encountered - Explore control and pain - Explore implications of engaging in activities/management individual has discussed as challenging   Explore emotional factors e.g fear *(Automatic motivation)*   - When an individual does x how does this make them feel (informed by interview of things that feel challenging – hobby, work, social) - Concerns about doing x activity with pain   Explore environmental factors and resources (*Physical Opportunity)*   - To what extent do physical or resource factors hinder x [e.g. pain management/ engagement in tasks/healthcare access for pain] - What things support/what make difficult   Probe: access, finance, time, information |
| The role of healthcare professionals | - Experience of healthcare - Healthcare professionals’ involvement with pain management | Experiences of healthcare since the last interview, has anything changed, new experiences  Explore social influences (*Social Opportunity)*   - Extent engagement with health and care professionals facilitated or hindered pain management |
| Other sources of support | - Family/friends - Information/materials/resources - Medium of support | Explore social influences (*Social Opportunity)*   - Extent engagement with family, peers, social groups facilitate or hinder pain management |

Italicised words are for researcher information. These map to the areas of the COM-B questionnaire.
