# Supplementary material for: Living with pain and Parkinson’s developing an understanding of the impact, trajectory and pain management needs: a qualitative interview study protocol
Source: BMJ Open. 2024 Dec 26;14(12):e078754. doi: 10.1136/bmjopen-2023-078754 (PMC11683903; doi:10.1136/bmjopen-2023-078754)
Supplement: Supplementary file 2 [file bmjopen-2023-078754.reviewer_comments.pdf]

PEER REVIEW HISTORY

BMJ Open publishes all reviews undertaken for accepted manuscripts. Reviewers are asked to complete a checklist review form and are provided with free text boxes to elaborate on their assessment. These free text comments are reproduced below.

ARTICLE DETAILS

Title (Provisional)

Living with pain and Parkinson’s: developing an understanding of the impact, trajectory, and pain management needs: A qualitative interview study protocol.

Authors

Naisby, Jenni; Avery, Leah; Baker, Katherine; Parkinson, Mark; Hand, Annette; Rochester, Lynn; Yarnall, Alison J; Walker, Richard William; Flynn, Darren; Ryan, Cormac; Finch, Tracy

---

VERSION 1 - REVIEW

**Reviewer**	**1**
**Name**	**Malini, Hema**
**Affiliation**	**Andalas University, Faculty of Nursing**
**Date**	**25-Jan-2024**
**COI**	**There is no conflict of interest to declare**

The authors need to clearly state why this study is important-- some of part in the introduction is really hard to understand, it didn't present in systematic ways

References need some work

VERSION 1 - AUTHOR RESPONSE

**Some of part in the introduction is really hard to understand, it didn't present in systematic ways**	Thank you for your comment.<br>Linking words have been added into the introduction to help sign post between key points.
**References need some work**	I have confirmed that all in-text references correspond with the reference list.
**One suggestion I have is to consider modifying the study design to better capture the nuances of pain experience in individuals with Parkinson's disease. Given that the study has not yet commenced, I recommend dividing participants into two groups: those with pain and those without pain.**	Thank you for your suggestion. We did not recruit people without pain in this study due to the aim to explore the in-depth experience of pain. Within the experience of pain, we expect to find great variation and therefore chose to focus solely on these individuals.
**Additionally, individuals with pain could be categorized based on their pain intensity, which is a critical aspect of pain management. A relevant study published in 2021 categorized the intensity of pain in Parkinson's patients, and I suggest incorporating this article into the methodology. Furthermore, it would be beneficial to include a pain screening component in the criteria for participant selection. This would enable the study to better capture the diverse experiences of individuals with Parkinson's disease and their corresponding pain management needs.**	Thank you for this suggestion.<br><br>We did not categorise pain in advance as we wanted to capture the subjective experience of pain from people living with Parkinson's. Whilst measures of intensity and categorisation are helpful, they do not capture the biopsychosocial impact of living with pain. An individual may experience pain of a lower severity but have high pain interference, lack of control, and wide ranging psychological impact. We chose to allow people who self-reported experiencing pain to participate and did not want to restrict this in any way. However, our inclusion criteria stated pain must interfere with daily life (page 7, line 28).<br><br>The severity and frequency of pain has been captured within the interviews and will be discussed in the results.
**- Sampling "PwP who experience pain" - please specify whether you are speaking about PD-related pain only.<br>- please clarify whether you will include also PwP who have pain due to other, non-PD related reasons (e.g. osteoarthritis). How will you clarify whether pain is PD-related or not?<br>- I suggest use of validated assessment tools to quantify PD pain and distinguish between its different subtypes.**	Thank you for your helpful comments.<br><br>Any individuals living with Parkinson's and who have experienced pain for at least three months that interferes with daily life (page 7, line 28) were eligible for participation.<br><br>We have not differentiated between PD-related and non PD-related pain for recruitment. Within the interview we explore

	when the pain started, the description of pain, if this has changed since diagnosis, and the influence (if any) of Parkinson's symptoms. We will be able to discuss our findings in the context of wider literature around the range of approaches of classifying pain in Parkinson's as appropriate.<br><br>Whilst we have also collected if individuals have been advised they have other painful conditions, Osteoarthritis for example, their pain experience is still relevant to Parkinson's due to the potentially altered nociceptive processing due to Parkinson's processes. Alongside this, understanding of how people manage living with pain and Parkinson's is important. It is also difficult to fully disentangle if pain is influenced by Parkinson's processes or not. As degeneration related to Parkinson's affects systems involved in the processing of pain, all pain could be influenced by these disease processes. This qualitative study aims to explore in detail the impact of living with pain and Parkinson's.<br><br>We have captured with people with Parkinson's the information they have received from healthcare professionals regarding the cause or classification of their pain and their own experiences and understanding of this. Whilst we understand the merits of a validated assessment tool, within the study we aimed to capture the multifactorial subjective experience of pain in individuals own words and chose not to classify this in advance. We have included reference to the important point of validated assessment on page 6 of the protocol.<br><br>Thank you for highlighting this important area and we will ensure to discuss this within the write up of the publication and consider recommendations for future use.

VERSION 2 - AUTHOR RESPONSE

Comment	Response

Please revise the title so that colons are used only once. E.g. Developing an understanding of the impact, trajectory, and pain management need when living with pain and Parkinson's: a qualitative interview study protocol.	This has been revised, thank you.
Given that the authors have already started the interviews, we would recommend including the interview guide as supplementary material	This has been included, thank you.
Despite the early stage of the work, we would recommend following the SRQR checklist for reporting of qualitative research and, if possible, include a copy as supplementary material indicating the page/line numbers of your manuscript where the relevant information can be found (http://journals.lww.com/academicmedicine/fulltext/2014/09000/Standards_for_Reporting_Qualitative_Research___A.21.aspx). Given that this is a protocol, the authors could indicate "N/A" for items that are not relevant	This has been followed where possible and will be fully reported in the completed published study.
